# Characterizing the Proliferation Patterns of Representative Microsporidian Species Enlightens Future Studies of Infection Mechanisms

**DOI:** 10.3390/pathogens11111352

**Published:** 2022-11-15

**Authors:** Jian Luo, Hailong Gao, Jinzhi Xu, Chen Xu, Tian Li, Zeyang Zhou

**Affiliations:** 1 State Key Laboratory of Silkworm Genome Biology, Southwest University, Chongqing 400715, China; 2 Chongqing Key Laboratory of Microsporidia Infection and Control, Southwest University, Chongqing 400715, China; 3 College of Life Science, Chongqing Normal University, Chongqing 400047, China

**Keywords:** *Nosema bombycis*, *Encephalitozoon hellem*, probes, life cycle, infection pattern

## Abstract

Background: Microsporidia are a group of pathogens that infect all kinds of animals, such as humans, silkworms, honeybees, and shrimp; they, therefore, pose a severe threat to public health and the economy. There are over 1500 species of microsporidia that have been reported, among which *Encephalitozoon hellem* and *Nosema bombycis* are the representative zoonotic and insect-infecting species, respectively. Investigating their cell infection patterns is of great significance for understanding their infection mechanisms. Methods: Specific probes were designed for the ribosomal RNA sequences of microsporidia. Fluorescence in situ hybridization (FISH) was used to trace the proliferation cycle of the pathogens in different cells. Results: Here, two rRNA large subunit gene (LSUrRNA) probes specifically labeling *N. bombycis* were obtained. The life cycle of *N. bombycis* in silkworm cells and *E. hellem* in three kinds of host cells was graphically drawn. *N. bombycis* meronts were first observed at 30 hours post-infection (hpi), and they began merogony. Sporonts were observed at 42 hpi, and the first entire proliferation cycle was completed at 48 hpi. The proliferation cycle of *E. hellem* in RK13 and HEK293 epithelial cells was almost the same, completing the first life cycle after 24 hpi, but it was significantly delayed to 32 hpi in RAW264.7. Conclusions: Specific FISH probes were established for labeling microsporidia in multiple host cells. The proliferation characteristics of representative zoonotic and insect-infecting microsporidian species were clarified. This study provides an experimental pattern for future analyses of microsporidian infection mechanisms.

## 1. Introduction

Microsporidia are obligate intracellular pathogens that infect a wide variety of animal hosts. *Nosema bombycis* infects silkworms via both horizontal and vertical transmissions, making it the causative agent of pébrine disease and a significant threat to sericulture worldwide [1,2,3]. *Encephalitozoon hellem* is a zoonotic microsporidian species that is frequently identified in avian hosts, livestock, and even humans. *E. hellem* infections cause keratoconjunctivitis, nephritis, and diarrhea in humans and could be fatal in immunocompromised individuals [4,5,6,7].

All microsporidian species share a general life cycle. It could be divided into the following three phases: the extracellular invasive phase, the proliferative (vegetative) phase, and the sporogonial (generative) phase [8,9]. During the infection phase, the microsporidian spores germinate and extrude a polar tube, which can penetrate host cell membranes and enable the transport of infective sporoplasms into the host cells [10]. The intracellular meront has a single-layer membrane structure, but there is no clear outline under white light, making it difficult to observe. The generative stage includes sporonts (cells that can produce two or more sporoblasts), sporoblasts (cells that are transformed into spores), and spores. A sporont is not only the initial cell at this stage of development but also the transitional cell that enters the sporogonic phase [11]. In *E. hellem*, all of the intracellular processes occur in the parasitophorous vacuole (PV). The number of nuclei and the duration of the intracellular developmental phases of the microsporidian life cycle are variable [11]. As a result, the details of the proliferating patterns of microsporidia are still unclear.

Microsporidian infections are often determined by detecting their spores [12]. The mature spores could be observed by phase-contrast microscopy or differential interference microscopy. Calcofluor White M2R and other fluorescent dye treatments [13,14,15] or more traditional stains, such as Giemsa, Gram, and Gram chromotropic acid stains, are all applicable for observing spores under a microscope [16,17]. The samples are usually prepared from silkworm eggs, feces, or other body fluid samples. However, these methods cannot distinguish the proliferation stages of the microsporidian cells. Conversely, transmission electron microscopy (TEM) has been proven to be an excellent tool for visualizing most of the structures and proliferation stages of microsporidia, especially the intracellular developmental stages, which are difficult to identify and differentiate [18]. Combined with some relatively new techniques, these traditional methods can identify the unique structures of microsporidia. However, the TEM has the disadvantage of being complicated to use and time-consuming. Recently, a fluorescent labeling method was used to label the proliferative phase of *N. bombycis* using a β-tubulin antibody [19]. Moreover, fluorescence in situ hybridization (FISH) was developed to detect and localize intracellular pathogens [20,21,22], including the microsporidia *Nematocida parisii* and *Nematocida displodere* [18,23,24]. The morphology and developmental characteristics of *N. bombycis* and *E. hellem* had to be observed by the traditional methods TEM, scanning electron microscope (SEM), and Giemsa [8,25,26,27]. However, none of the above studies can directly and consistently show the proliferation cycle of microsporidia in host cells, and there is no comparison of the proliferation patterns of the pathogens in different hosts.

In this study, by utilizing the representative *N. bombycis* and *E. hellem* species, we used FISH methods to precisely characterize the life cycle of the parasites. Our study aims to provide an experimental pattern for revealing microsporidian infections and help with other studies on the biology and gene functions of microsporidia.

## 2. Materials and Methods

### 2.1. Purification of Microsporidian Spores

The microsporidian *Nosema bombycis* CQ 1 was isolated from the silkworm *Bombyx mori* in Chongqing, China (102059, CVCC). *Encephalitozoon hellem* was gifted by Dr. Han Bing from the Shandong University, China. Heavily infected silkworm pupae were removed from the skin and degenerated midgut. The pupa tissues were gently homogenized in physiological saline and filtered using a 10 mL centrifuge tube containing thick cotton. The spores were purified by centrifugation at 10,000× *g* for 20 min in 90% Percoll (17089102, Cytiva). The purified spores were washed with sterile water three times, suspended in 0.5 mL of water, and, finally, stored at 4 °C for one month [28]. *E. hellem* spores were collected from infected rabbit kidney cells (RK13) (CCL37, ATCC) and purified by centrifugation at 10,000× *g* for 15 min in 75% Percoll to collect the lowest pathogen [29]. In this research, we used fresh spores for each infection.

### 2.2. Cell Infections with Spores of N. bombycis and E. helllem

Silkworm *Bombyx mori* embryonic cells (BmEs) were cultured in Grace’s medium supplemented with 10% fetal bovine serum (FBS) (10100154, Gibco, Thermo Fisher Scientific) at 28 °C. The BmE cells were added to a 12-well plate at 5 × 10^5^ cells per well. The purified *N. bombycis* spores were treated with 0.1 M KOH for 3 min and then added to the wells at a final ratio of 30:1 spores/cell [30,31,32]. The infected cell samples were prepared at 3, 6, 9, 12, 18, 24, 30, 36, 42, 45, 48, 54, 60, 66, 72, 80, 96 h post-infection (hpi), respectively.

RK13 (ATCC, CCL-37), human embryonic kidney (HEK293) (ATCC, CRL-1573), and macrophage (RAW264.7) (ATCC, TIB-71) cell lines were cultured and grown in complete growth medium supplemented with 10% FBS at 37 °C with 5% CO_2_. The cells were infected with the *E. hellem*:cells = 30:1 [29,33,34]. The cell samples were prepared at 4, 8, 12, 16, 20, 24, 28, 32, 36, 40, 48, and 56 hpi.

### 2.3. FISH Probes for Labeling the Microsporidia

The complete sequence of the ribosomal RNA (rRNA) and the secondary structure model of the rRNA large subunit gene (LSUrRNA) of *N. bombycis* were obtained [35]. According to the *N. bombycis* rRNA complete sequence, we designed nine rRNA probes and synthesized them with a Quasar 570 (Cy3) 5′ modification with high-performance liquid chromatography purification by Sangon Biotech Co., Ltd. (Shanghai, China) (Table 1). In addition, a Cy3-ACTCTCACACTCACTTCAG probe (HEL878F probe) had been reported for the specific labeling of *E. hellem* [20,36].

### 2.4. FISH Staining

The infected cells were fixed with 4% paraformaldehyde for 20 min and washed with phosphate-buffered saline (PBS) three times, followed by incubation overnight at 46 °C with hybridization buffer (900 mM NaCl, 20 mM Tris [pH 7.5], and 0.01% sodium dodecyl sulfate [SDS]) containing 5 ng/µL of the probes. The infected cells were then washed at 48 °C for 1 h in wash buffer (900 mM NaCl, 20 mM Tris [pH 7.5], 0.01% SDS, and 5 mM ethylenediaminetetraacetic acid) [24], after which they were washed in PBS three times and then stained in DAPI (4′6-diamidino-2-phenylindole, Sigma) for 30 min. After three additional washes with PBS and the addition of ProLong1 Gold anti-fade reagents, the infected cells were observed using Olympus FV1200 laser scanning confocal microscopy. With the photos, we randomly selected 10 visual fields, and the ratio of infected cells in each field was calculated by manual counting. The lengths and widths of the parasites were determined using ImageJ 1.52v (https://imagej.nih.gov/ij/index.html, accessed on 13 April 2020), and they were analyzed for a *t*-test with GraphPad Prism v6.01 (https://www.graphpad.com/scientific-software/prism/, accessed on 21 September 2012). The experiments were conducted at least three times.

## 3. Results

### 3.1. Pathogen Infections Demonstrated by FISH

**Figure 1:**
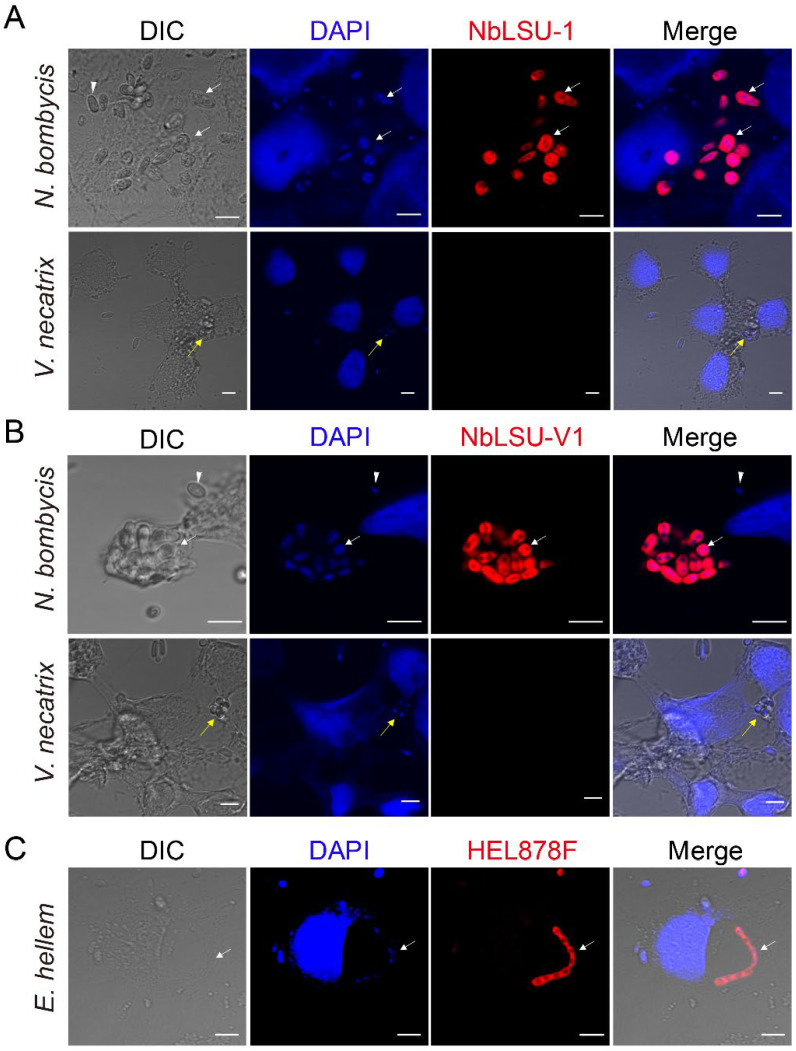
The specificity of the NbLSU-1 (**A**) and NbLSU-V1 (**B**) probes was detected in *N. bombycis*- and *V. necatrix*-infected BmE cells, respectively. The specificity of the HEL878F probes (**C**) was detected in *E. hellem*-infected RK13 cells. Laser confocal microscopy showed a nucleus labeled with DAPI (blue). The microsporidia were labeled with the rRNA FISH probe (red). The white arrow indicates FISH staining of multinucleate microbes (proliferative or sporogonial phases); the yellow arrow indicates *V. necatrix* xenoma; the arrowhead indicates the mature spores. Scale bar, 5 µm.

We designed and tested nine probes. The results showed that there are two rRNA-specific fluorescence in situ hybridization (FISH) probes NbLSU-V1 and NbLSU-1 that could specifically label *N. bombycis* meronts inside BmEs but not *Vairimorpha necatrix* (Figure 1A,B). The NbLSU-V1 probe was chosen for the subsequent experiments. On the other hand, *E. hellem* meronts inside host cells could be labeled by the rRNA-specific HEL878F probe (Figure 1C). The FISH probes can label meronts, sporonts, and sporoblasts, but not mature spores. Thus, the combined application of DAPI and FISH can distinguish pathogens in developmental stages from dormant spores.

### 3.2. The Proliferation and Life Cycle of N. bombycis in BmE Cells

**Figure 2:**
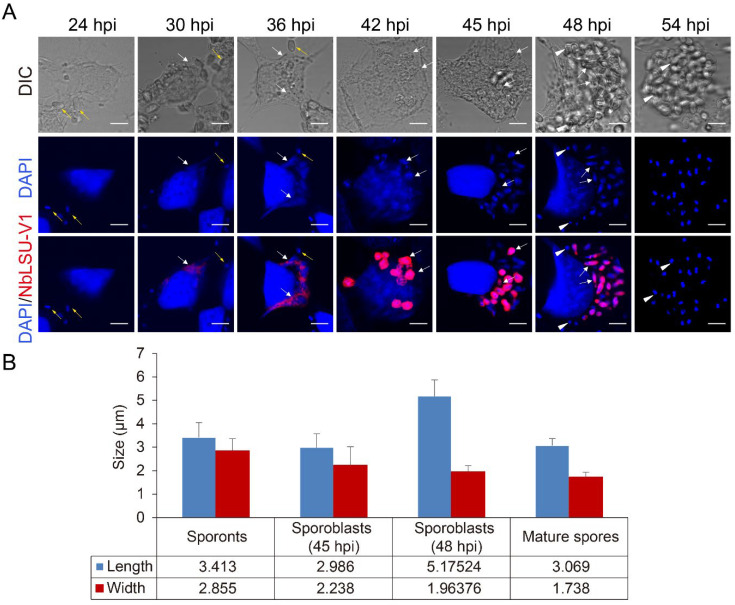
(**A**) The life cycle of *N. bombycis* in BmE cells. The probe was added to the infected cells at the following time points: 24, 30, 36, 42, 45, 48, and 54 hpi. The blue and red fluorescent signals represent nuclear DNA and pathogens at different stages, respectively. The white arrow represents FISH staining of the proliferating spores in the intracellular phase; the yellow arrow shows the spores that may be extracellular or attached to the cell surface; the arrowhead indicates the mature spores; scale bar, 5 µm. (**B**) At least 26 spores’ lengths and widths were calculated during the sporogony phase. The y-axis indicates the spore size; the x-axis indicates each stage of the spores; the average size values are shown.

A typical time course of an *N. bombycis* infection and proliferation within host cells is illustrated in Figure 2. As shown, no pathogens were detected by 24 hpi. By 30 hpi, the parasites were detected by FISH staining for the first time, indicating that they were in merogony (Figure 2A). The meronts gradually grew larger and became irregular in shape until they occupied about a quarter of the host cytoplasm. The nuclei were irregularly shaped and less compact, possibly suggesting that the chromosomes were replicating. By 42 hpi, sporogony was observed. The sporonts were bounded by a typical plasma membrane and were fairly round in shape, with 1.7–2.8 µm in width and 3 µm in length on average (Figure 2B). The two sets of paired nuclei of sporonts were clearly visible. By 45 hpi, the parasites were in the sporoblast stage and elongated up to 7 µm in length, each containing two nuclei (Figure 2 and Figure S1). By 48 hpi, some pathogens had completed sporulation and produced mature spores, which could not be labeled by the probe. Finally, a massive production of mature spores was observed after 54 hpi (Figure 2).

**Figure 3:**
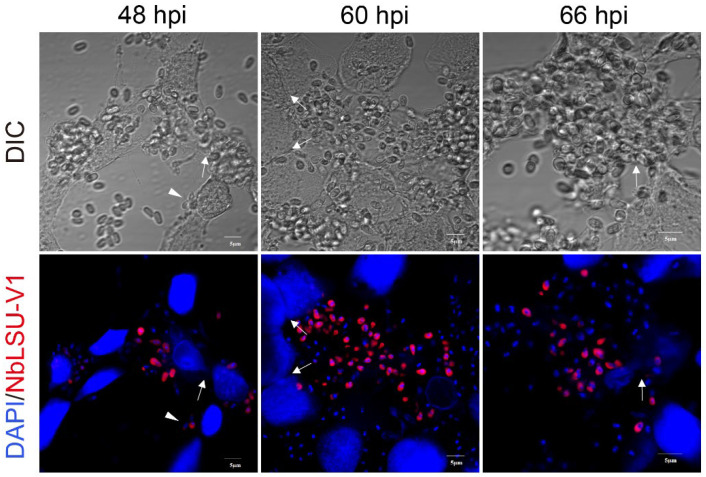
Syncytia formation between adjacent infected cells. The infected BmE cells after 48, 60, and 66 hpi were stained with DAPI (nucleus, blue) and the rRNA FISH probe (red). The arrow indicates the syncytia between the infected cells. The arrowhead indicates the secondary infective form.

Additionally, some adjacent host cells were found to fuse with each other after 48 hpi (Figure 3). Host cell fusion was also observed in *Nematocida parisii*-infected *Caenorhabditis elegans* [23]. Therefore, in BmEs, *N. bombycis* begins to proliferate at 30 hpi and completes the first round of life cycles at 48 hpi, after which the pathogen probably starts the secondary infections.

### 3.3. The Proliferation and Life Cycle of E. hellem in RK13, HEK293, and RAW264.7 Cells

**Figure 4:**
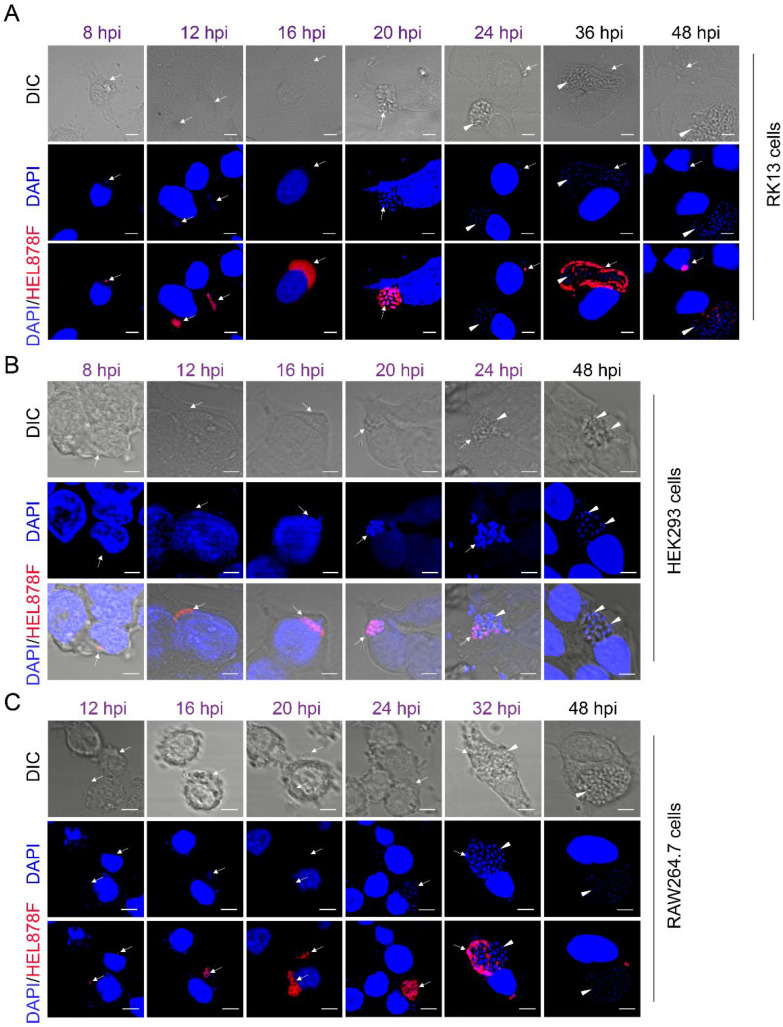
The life cycle of *E. hellem* in RK13 (**A**), HEK293 (**B**), and RAW264.7 (**C**). Laser confocal microscopy showed a nucleus labeled with DAPI (blue). The pathogens were labeled with the FISH probe (red). The arrow represents FISH staining of the proliferating spores in the intracellular phase. The arrowhead indicates the mature spores; scale bar, 5 µm.

It was found that the proliferation cycles of *E. hellem* in RK13 and HEK293 were identical. At 8 hpi, the pathogen was labeled with the FISH probe for the first time in both cells (Figure 4A,B). Multinucleated meronts were observed at 12 hpi, which were thin or irregular and located at the edge of the plasma membrane. At 16 hpi, the volume of the pathogen was further expanded, the shape was more irregular, and the number of nuclei significantly increased, but the pathogen outline was still not visible at this time, indicating they were also in the proliferative phase. At 20 hpi, sporogony was observed, and the parasitophorous vacuole (PV) was visible in the DIC. The mature spores were visible after 24 hpi, and they were detected with UV light but not with the FISH probe (Figure 4A,B). In the case of the RK13 and HEK293 cell infections, *E. hellem* invaded the host cells after 8 hpi and began to proliferate, followed by the proliferative phase at 8–16 hpi and sporogonial stage at 16–20 hpi, and finally completed a life cycle round at 24 hpi.

In comparison, the *E. hellem* meronts in the RAW264.7 cells appeared after 12 hpi. At 24 hpi, the spores gradually formed the spore wall in the RAW264.7 cells. After 32 hpi, a round of proliferation in RAW264.7 was completed. In general, the proliferation cycle of *E. hellem* in RAW264.7 was delayed by about 8 h compared with that in RK13 and HEK293 epithelial cells (Figure 4C).

Our results also demonstrated that an *E. hellem* infection was not completely synchronous. At 36 hpi, a larger PV was observed, and the mature spores in the inner layer, while in the outer layer, were still in the stage of meronts (Figure 4A), which further confirms that the outer edge meronts of PV were more favorable to exchange substances. At 24 and 48 hpi, the developmental stages were uneven, and we could observe the co-existence of many mature pathogens and meronts in different cells, possibly related to the secondary infection of the spores (Figure 4A).

### 3.4. The Infection Patterns of N. bombycis and E. hellem

**Figure 5:**
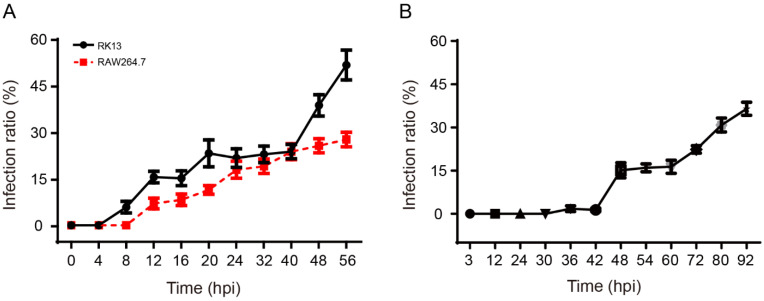
The infection patterns of *N. bombycis* and *E. hellem* in host cells. (**A**) The proliferation and life cycle of *E. hellem* in RK13 and RAW264.7 cells. (**B**) The proliferation and life cycle of *N. bombycis* in BmE cells.

Next, we calculated the percentage of infected cells at each sampling time. The infection rate increased in RK13 and RAW264.7 and synchronously reached 23% at 40 hpi. This implied that most pathogens had completed their first life cycle at this time (Figure 5A). Furthermore, the infection rate increased sharply after 40 hpi in RK13, but the infection was always relatively slower in RAW264.7, suggesting that the infection rate of *E. hellem* was significantly lower in RAW264.7 than that in RK13. This discrepancy may be caused by the different mechanisms of pathogen infection and immune pressure.

Similarly, we analyzed the pattern of *N. bombycis* in BmE cells. We found that the infection rate did not significantly change until 42 hpi, but a rapid increase was detected after 42–48 hpi, implying that most pathogens had completed the initial infection phase and began the proliferate phase at 42 hpi. Subsequently, the infection rate stayed at a certain higher level from 48 to 60 hpi, suggesting that the rates of parasite infection and host cell proliferation had achieved an equilibrium. After 60 hpi, however, the infection rate sharply increased to about 40%, indicating that the pathogens quickly multiplied by the second or third round of infection (Figure 5B).

## 4. Discussion

Studies on microsporidia have been carried out for more than 150 years. To observe their infections in situ, different staining methods were developed [8,19,24,37]. Among which, fluorescence in situ hybridization (FISH) shows comparative advantages in resolution and usability. As shown in this study, nearly all of the stages of the life cycles of *N. bombycis* and *E. hellem* were labeled, except for the mature spores.

A total of nine probes were designed, which were derived from the small subunit (SSU) rRNA and internal transcribed spacer (ITS) region, as well as the linear and hypervariable areas of the secondary structure LSU rRNA of *N. bombycis* [35]. However, only the probes from the linear (NbLSU-1) and hypervariable regions (NbLSU-V1) were well labeled. This phenomenon was also found in the relative fluorescence intensity analysis of the oligonucleotide probes for the model 16S rRNA secondary structure in *Escherichia coli* [38]. Although the *E. hellem* HEL878F probe had been reported, the FISH protocol was completely different from the previous one. In summary, the current study provides an effective and useful method for precisely tagging the microsporidian life cycle. Although the life cycle of *E. hellem* has been explored in a single host cell [25,26,36,39,40], our study compared *E. hellem* proliferation in multiple cells for the first time. The proliferation of *E. hellem* in the macrophage cell line showed up 8 h later than that in the epithelial cell line, which may be caused by the immune pressure of macrophages. It is known that macrophages are the intermediate link between innate immunity and adaptive immune response and are essential for the early invasion of pathogens [41]. In addition, macrophages are reported to recognize microsporidian infections via toll-like receptors (TLR2) and the release a large number of defense mediators, such as IFN-γ and IL-12, against other intracellular viruses, bacteria, and parasites [41,42]. The discrepancy in the proliferation patterns is also reported in the infection cycle of *Toxoplasma gondii*, which showed a 2.6 h delay in HeLa cells compared with bovine embryonic skeletal muscle cells [43,44].

The proliferation pattern of *N. bombycis* used to be studied by Giemsa staining and TEM and was found to be variable between *Bombyx mori* ovarian (BmN) and Antheraea eucalypti cells [8,37,45,46,47,48]. In A. eucalypti cells, *N. bombycis* sporoplasms were initially observed after 6 hpi and developed to meronts by 18 hpi, and the mature spores were present after 42 hpi [49]. *N. bombycis* were found to develop slower in BmN cells, in which the sporoblasts appeared after 54 hpi, the mature spores were observed after 60 hpi, and the massive mature spores were formed by 96 hpi [27]. In our study, the *N. bombycis* life cycle in BmE cells is shorter than that of BmN but longer than that of A. eucalypti cells. None of these cells are immune cells; the reason for the discrepancy is unclear. However, pathogen adaptation, dosage of infection, culture environment, and different staining methods are probably important factors.

Moreover, it is worth mentioning that the FISH probes could specifically identify meronts, sporonts, and sporoblasts. Our study provides a reference for the selection of appropriate cell lines for future *E. hellem* infection studies, as well as for the determination of the infection time in different cell lines. Additionally, the current study is based on in vitro cell cultures and does not implicate the adaptive immunity of the host. For future studies, we will expand our investigations to different cell lines and tissues to provide a better understanding of microsporidian proliferation patterns within hosts.

## 5. Conclusions

In summary, our study verified two FISH probes for specific microsporidian labeling, characterized the proliferation patterns of the representative microsporidian species *N. bombycis* and *E. hellem* for the first time, and enlightened future investigations of microsporidian infection mechanisms.

## Figures and Tables

**Table 1:** The probe sequences of *N. bombycis* rRNA.

Probe Name	Probe Sequence (5′-3′)
NbLSU-1	GAACATTAGGTTTCTATCCT
NbLSU-2	TTGTATCTTAGGACAACTGTG
NbLSU-V1	GCAATCGTACTCTACATTG
NbLSU-V3-D3	TACTGTCATCTGGTAATCT
NbLSU-V4-D13	CGCCCACTTGAGTATCGT
NbLSU-V4-E14	TAACAACTATCACATCATAT
NbLSU-I3	TCATTCTTACAGTCCCACTC
NbSSU	CCTGGTAAATTACCCCGCG
NbITS	TTACCCCGCGTTGAGTCAAA

## Supplementary Materials

The following supporting information can be downloaded at: https://www.mdpi.com/article/10.3390/pathogens11111352/s1, Figure S1: *N. bombycis* sporoblasts at 48 hpi. Laser confocal microscopy showing a nucleus labeled with DAPI (blue). The pathogens were labeled with the FISH probe (red). Scale bar, 5 µm.
