# Supplementary material for: Characterizing the Proliferation Patterns of Representative Microsporidian Species Enlightens Future Studies of Infection Mechanisms
Source: Pathogens. 2022 Nov 15;11(11):1352. doi: 10.3390/pathogens11111352 (PMC9692852; doi:10.3390/pathogens11111352)
Supplement: Supplementary file 1 [file pathogens-11-01352-s001.zip › pathogens-1998070-supplementary.pdf]

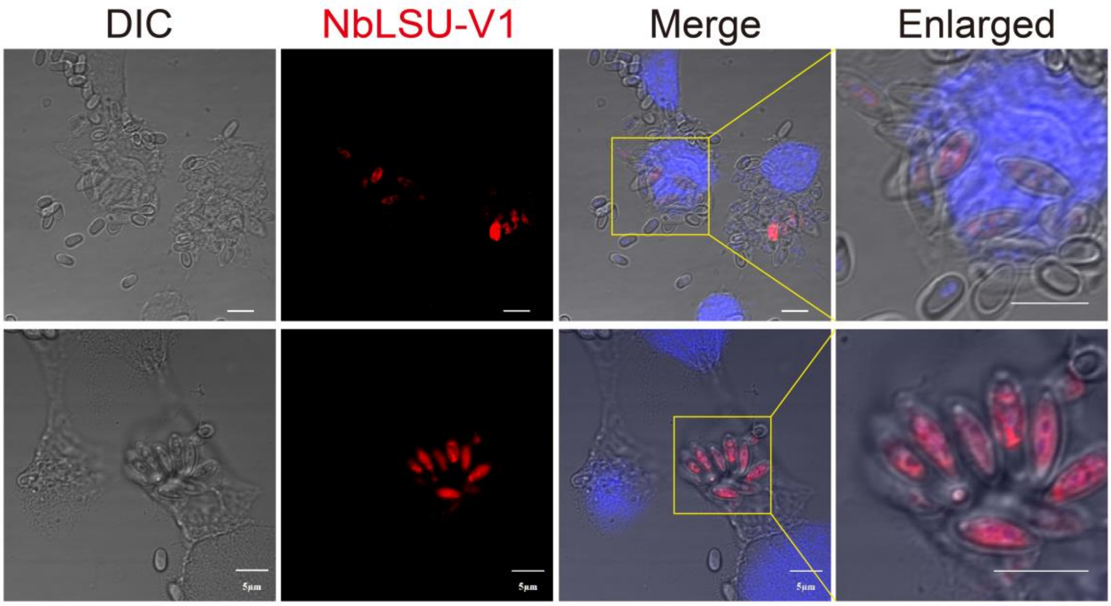

Figure S1. *N. bombycis* sporoblasts at 48 hpi. Laser confocal microscopy showing a nucleus labeled with DAPI (blue). The pathogens were labeled with the FISH probe (red). Scale bar, 5 μm.
